# Image Reconstruction for Diffuse Optical Tomography Based on Radiative Transfer Equation

**DOI:** 10.1155/2015/286161

**Published:** 2015-01-14

**Authors:** Bo Bi, Bo Han, Weimin Han, Jinping Tang, Li Li

**Affiliations:** ^1^Department of Mathematics, Harbin Institute of Technology, Harbin, Heilongjiang 150006, China; ^2^School of Mathematics and Statistics, Northeast Petroleum University, Daqing, Heilongjiang 163318, China; ^3^Department of Mathematics, University of Iowa, Iowa City, IA 52242, USA; ^4^Imaging Diagnosis and Interventional Center, State Key Laboratory of Oncology in South China, Sun Yat-sen University Cancer Center, Guangzhou, Guangdong 510060, China

## Abstract

Diffuse optical tomography is a novel molecular imaging technology for small animal studies. Most known reconstruction methods use the diffusion equation (DA) as forward model, although the validation of DA breaks down in certain situations. In this work, we use the radiative transfer equation as forward model which provides an accurate description of the light propagation within biological media and investigate the potential of sparsity constraints in solving the diffuse optical tomography inverse problem. The feasibility of the sparsity reconstruction approach is evaluated by boundary angular-averaged measurement data and internal angular-averaged measurement data. Simulation results demonstrate that in most of the test cases the reconstructions with sparsity regularization are both qualitatively and quantitatively more reliable than those with standard *L*_2_ regularization. Results also show the competitive performance of the split Bregman algorithm for the DOT image reconstruction with sparsity regularization compared with other existing *L*_1_ algorithms.

## 1. Introduction

Diffuse optical tomography (DOT) is an emerging imaging modality that has attracted much attention in clinical diagnosis, for example, in breast cancer detection, monitoring of infant brain tissue oxygenation level, and functional brain activation studies, cerebral hemodynamic, and so forth; compare [1–3]. With the use of near-infrared (NIR) light, DOT probes the optical properties, mainly the absorption coefficient and the scattering coefficient of human tissues. In experimental systems, a set of optical fibres and optodes are attached to the boundary of the object as measurement detectors and sources. NIR light as the inflow current is emitted by laser and guided by some fibre optics into the object, one position at a time. The light is transmitted, and then the outflow current is measured from all the measurement positions using light sensitive detectors.

By employing a setup comprising a set of external light sources and detectors, the optical properties of a tissue can be recovered by applying the principles of tomography. The difference in absorption or scattering between the normal and abnormal tissues can provide the imaging contrast for tissue diagnostics. In this context, the aim of diffuse optical tomography is to provide the spatial distribution of the absorption and scattering coefficients of a tissue.

The forward problem in DOT describes the photon propagation in tissues and the inverse problem involves estimating the absorption and scattering coefficients of tissues from light measurements on the surface. There are many models describing the light propagation, for example, the Fokker-Planck equation, differential approximations of the radiative transfer equation (RTE) and so forth (cf. [4, 5]), and the diffusion equation (DA), which is accepted as a popular forward model describing light propagation in tissues. DA is a low-order approximation of RTE. However, the validation of DA breaks down in the following situations. First, since DA is only valid in the tissues with high scattering and low absorption, while in many tissues of human body with low scattering, such as skeleton, joint, DA is largely limited. Second, DA is not suitable to describe the light propagation in regions near to the light source, which will result in the large error when using DA as the forward model to image small animal or small tissue. For more on DA, we refer to [1, 6, 7]. Due to these limitations of DA, RTE is a more natural choice to model the light propagation. In this paper, we will present numerical evidence to show that DOT based on RTE can achieve satisfactory resolution.

Since DOT suffers from severe ill-posedness caused by noise and incomplete measurement data, its efficient, stable, and accurate numerical treatment is very challenging. Due to its immense range of prospective applications, there has been a vast amount of research work on mathematical as well as practical aspects of the inverse problem. In particular, the design of efficient and stable numerical algorithms has received considerable attention.

In the linearized Jacobian-based methods, a popular approach for solving the minimization problem is to treat it as a nonlinear least-square problem that can be solved with many standard optimization techniques [8], among which Levenberg-Marquardt method (LM) [9] is perhaps the most commonly used one, which is a Gauss-Newton method with *L*_2_ regularization. Based on RTE, the authors in [10] discussed in detail the LM method for parameter estimation problems. We will describe the LM method briefly in Section 3. In the nonlinear gradient-based methods, we directly minimize the nonlinear functional; in the optimization process only the gradient of the nonlinear functional needs to be computed, for example, the BFGS (Broyden-Fletcher-Goldfarb-Shanno) method and the L-BFGS method [8, 11].

The appropriate choice of regularization depends on* a priori* knowledge of the domain and the inclusions. Although *L*_2_ regularization is usually a natural choice for its simplicity, it is not the optimal strategy. For example, when the coefficient distribution is sparse or discontinuous, it is well known that the *L*_1_ regularization method [12, 13] or the bounded total variation (TV) regularization method [14, 15] is more efficient than *L*_2_ regularization.

Sparse reconstruction has attracted much researchers' attention, especially since Donoho et al. [16, 17] established the theory of compressive sensing (CS). CS describes the sparse reconstruction problem as an *L*_0_ quasinorm optimization [18]. However, Donoho in [19] proved that the *L*_1_ regularization can also obtain the sparsest solution and proposed the equivalent condition between the *L*_1_ regularization and the *L*_0_ regularization. In [20], Candes et al. studied the stable signal recovery from incomplete and inaccurate measurements and reduced a computationally difficult problem to the basis pursuit problem. Because the sparsity regularization is nonsmooth, it is still a challenge to find efficient methods to solve this convex basis pursuit optimization problem, and the choice of techniques for solving it becomes crucial. Classical gradient-based methods usually bring high computational burden [21]. Motivated by the inverse problem in imaging [22–25], the authors used and developed the Bregman iteration technique for the *L*_1_ regularization problems and proved that the Bregman iteration method is an effective way to solve the *L*_1_ norm minimization problems.

In this paper, we adopt the split Bregman method for solving sparsity regularization problems. The split Bregman method is a simple and efficient algorithm which can split the minimization into *L*_1_ and *L*_2_ functionals and significantly reduce the computational burden [24]. We will introduce the split Bregman algorithm in detail in Section 3.

The organization of this paper is as follows.  Section 2 introduces the DOT forward problem, briefly presents the radiative transfer equation (RTE), and describes the imaging modality of the forward problem. Section 3 presents in detail the reconstruction algorithm, including the Levenberg-Marquardt algorithm and the split Bregman algorithm.  Section 4 provides several simulations to show the validity of the sparsity regularization reconstruction.  Section 5 gives some conclusion statements.

## 2. Forward Problem

In this section, we formulate our problem of RTE based diffuse optical tomography.

### 2.1. Radiative Transfer Equation

Photon propagation in tissues can be described by the radiative transfer equation. Let *X* ⊂ *R* ^*n*^, *n* = 2 or 3, denote the physical domain of the medium with boundary ∂*X*,  *Ω* : = *S* ^*n*−1^ the unit sphere, ***ν***(**x**) the unit outer normal vector, and Γ_±_ ⊂ ∂*X* × *Ω* the outgoing and incoming boundaries defined by

(1) $$\begin{array}{c} \Gamma_{+}=\left\{\left(x,\omega \right)\in \partial X\times \Omega \;|\;\omega \cdot \nu \left(x\right)>0\right\}\\ \Gamma_{-}=\left\{\left(x,\omega \right)\in \partial X\times \Omega \;|\;\omega \cdot \nu \left(x\right)<0\right\}. \end{array}$$

The variables **x** ∈ *X* and **ω** ∈ *Ω* denote the spatial position and the angular direction. Then we consider the following boundary value problem (BVP) of RTE:

(2) ω·∇ux,ω+μtxux,ω=μsxSux,ωM+f(x,ω) in  X×Ω,

(3) $$\begin{array}{c} u\left(x,\omega \right)=u_{\text{in}}\left(x,\omega \right)\;\mathrm{on}\;\;\Gamma_{-}, \end{array}$$

where *μ*_*t*_(**x**) = *μ*_*a*_(**x**) + *μ*_*s*_(**x**) is the total attenuation coefficient, *μ*_*a*_(**x**) describes the probability that a photon is absorbed in unit length, its reciprocal 1/*μ*_*a*_ being the absorption mean free path, and *μ*_*s*_(**x**) is the scattering coefficient, describing the probability that a photon is scattered in unit length, its reciprocal 1/*μ*_*s*_ being the scattering mean free path. Further, *u*(**x**, **ω**) is the radiance and *f*(**x**, **ω**) is the internal light source. In this paper, we consider the case with no light source inside *X*; *f*(**x**, **ω**) = 0.  *u*_in_(**x**, **ω**) is the inflow current on Γ_−_, and the boundary condition (3) implies that once a photon escapes the domain *X*, it does not reenter it; compare [26]. *S* is the scattering operator. Denote by *dσ*(**ω**) the infinitesimal area element on the unit sphere *Ω*. Then *S* is defined as

(4) $$\begin{array}{c} \left(Su\right)\left(x,\omega \right)=\int_{\Omega }\eta \left(x,\omega \cdot \hat{\omega }\right)u\left(x,\hat{\omega }\right)d\sigma \left(\hat{\omega }\right) \end{array}$$

with *η* a nonnegative normalized phase function:

(5) $$\begin{array}{c} \int_{\Omega }\eta \left(x,\omega \cdot \hat{\omega }\right)d\sigma \left(\hat{\omega }\right)=1\;\forall x\in X,\;\;\omega \in \Omega . \end{array}$$

In many applications, the function *η* is independent of **x**. However, in our general study, we allow *η* to depend on **x**. Indeed, we can even allow *η* to be a general function of **x**, **ω**, and $\hat{\omega }$, that is, in the form $\eta (x,\omega ,\hat{\omega })$. The scattering phase function $\eta (x,\omega \cdot \hat{\omega })$ describes the probability that a photon with an initial direction $\hat{\omega }$ will have a direction **ω** after a scattering event. In DOT, one typical example is the Henyey-Greenstein phase function; compare [27]. Consider

(6) $$\begin{array}{c} \eta \left(t\right)=\frac{1-g^{2}}{4{\left(1+g^{2}-2gt\right)}^{3/2}},\;t=\omega \cdot \hat{\omega }\in \left[-1,1\right], \end{array}$$

where the parameter *g* ∈ (−1,1) is the anisotropy factor of the scattering medium. Note that *g* = 0 for isotropic scattering, *g* > 0 for forward scattering, and *g* < 0 for backward scattering. In biomedical imaging problems, the scattering is strongly forward peaked and *g* is close to 1.

### 2.2. Forward Problem

Similar to the X-ray CT, DOT experiments acquire the current distribution of detectors on the boundary under the multi-incidents [28]. The experimental procedure of acquiring potential measurements is as follows. First, set a set of *s* laser devices and *d* detectors on the boundary of the object. Then launch an incident impulse from one laser device and record the resulting measurements from all the detectors. In order to gather enough data information, repeat this procedure on other laser devices.

We can model this procedure mathematically. Before doing this, let us introduce some assumptions on the domain and the coefficients, assumed to be valid throughout the rest of this paper.

We assume that the absorption and scattering coefficients are approximated piecewise-constant. It is known that the BVP (2) and (3) has a unique solution [29], and the solution depends continuously on the input current *u*_in_ on the incoming boundary Γ_−_.

The forward problem of DOT is to determine the outgoing current on the detectors when the incident impulse and the absorption and scattering coefficients are known. Excite the domain *X* with a sequence of incident impulses *u*_in,*i*_, 1 ≤ *i* ≤ *s*, and get a sequence of measurements *M*_*i*_ corresponding to each incident impulse, with its component *M*_*i*_^*j*^, 1 ≤ *j* ≤ *d*, being the measurement value on *d* detectors. Then a mathematical description of such an experiment is provided by a sequence of forward operators:

(7) $$\begin{array}{c} F_{i}:D⟶R^{d},\;\left(\mu_{t},\mu_{s}\right)⟼M_{i},\;1\leq i\leq s \end{array}$$

which maps prescribed optical parameters to the corresponding measurements. Here, *F*_*i*_  denotes the *i*th forward operator with respect to the *i*th incident impulse and the resulting detected measurement data on *d* detectors. Then, for 1 ≤ *i* ≤ *s*, the domain of the operator *F*_*i*_ is defined as

(8) $$\begin{array}{c} D=\left\{\left(\mu_{t},\mu_{s}\right)\in L^{\infty }\left(X\right)\times L^{\infty }\left(X\right)\right\}. \end{array}$$

*M*_*i*_ ∈ *R* ^*d*^ is a column vector representing the measurement data on *d* detectors.

We mention that, in DOT, the measured quantity is the excitance on the boundary of the domain. Due to the limit of measurement techniques of the optical devices, the angularly resolved measurement data *u*|_Γ_+__ is not practical. In this study, for *i* = 1,…, *s*, we use the boundary angularly averaged data as our measurements [30]:

(9) $$\begin{array}{c} M_{i}=\int_{\Omega_{x,+}}\omega \cdot \nu \left(x\right)u_{i}\left(x,\omega \right)d\sigma \left(\omega \right),\;x\in \partial X, \end{array}$$

where *u*_*i*_(**x**, **ω**) is the solution of the BVP (2) and (3) corresponding to the optical coefficients (*μ*_*t*_, *μ*_*s*_) and the *i*th incident impulse *u*_in,*i*_ and ***ν***(**x**) is the unit outer normal vector, whereas the set

(10) $$\begin{array}{c} \Omega_{x,+}:=\left\{\omega \in \Omega \;|\;\omega \cdot \nu \left(x\right)>0\right\}. \end{array}$$

In [30], a detailed analysis is given on properties of the forward operator, including the Lipschitz continuity and the* Fréchet* differentiability. The BVP (2) and (3) needs to be solved by numerical methods, such as the finite difference method or the finite element method in spatial discretization, and the finite element method or *S*_*N*_ approximation in angular discretization. For the simulation results in this paper, we use the RTE2DMATLAB codes [6], in which the finite element method in both spatial and angular spaces is used to discretize the forward mapping. We refer the reader to [4, 6, 31–34] for details about the finite element implementation in both spatial and angular space.

## 3. Inverse Problems

In practice, due to the limitations of the experimental environment and the laboratory equipment, the measurement *M*_*i*_ we get usually contains noise. Here, we assume that, for 1 ≤ *i* ≤ *s*, the actual measurements have the noise level *δ*; that is, ‖*M*_*i*_^*δ*^ − *M*_*i*_‖ ≤ *δ*, where *M*_*i*_^*δ*^ represents the actual measurement data and *M*_*i*_ represents the true data corresponding to the true optical coefficients. Then our inverse problem of DOT is to determine (*μ*_*t*_, *μ*_*s*_) such that the following nonlinear equations hold:

(11) $$\begin{array}{c} F_{i}\left(\mu_{t},\mu_{s}\right)=M_{i}^{\delta },\;\left(\mu_{t},\mu_{s}\right)\in D \end{array}$$

for *i* = 1,…, *s*.

In this paper, we only reconstruct the scattering coefficient, while assuming that the distribution of the total attenuation coefficient *μ*_*t*_ is known.

As is typical for many inverse problems, the DOT inverse problem is ill-posed. In order to reconstruct the optical parameter stably, regularization is required. We minimize the following Tikhonov functional:

(12) $$\begin{array}{c} J\left(\mu_{s}\right):=\frac{1}{2}\overset{s}{\underset{i=1}{\sum }}{\left\|F_{i}(\mu_{s})-M_{i}\right\|}_{L^{2}(\partial X)}^{2}+\alpha R\left(\mu_{s}\right), \end{array}$$

over the admissible set

(13) $$\begin{array}{c} Q_{\text{ad}}=\left\{\mu_{s}\in L^{\infty }\left(X\right)\right\} \end{array}$$

for the coefficient *μ*_*s*_. Here, *R*(*μ*_*s*_) is a regularization penalty functional that enforces* a priori* knowledge on the optical parameter to be reconstructed and *α* > 0 is the regularization parameter used to trade off the discrepancy term (the first item of *J*(*μ*_*s*_)) that incorporates the information contained in the data and *R*(*μ*_*s*_) [35]. Then we analyse the minimization problem:

(14) $$\begin{array}{c} \underset{\mu_{s}\in Q_{\text{ad}}}{\inf}J\left(\mu_{s}\right). \end{array}$$

We consider two popular regularization formulations: the *L*_2_ norm penalty and the sparsity constraint regularization. We will describe these two approaches below.

### 3.1. Standard Reconstruction

First, we use the traditional *L*_2_ norm squared penalty, which consists of minimizing the following functional:

(15) $$\begin{array}{c} J\left(\mu_{s}\right)=\frac{1}{2}\overset{s}{\underset{i=1}{\sum }}{\left\|F_{i}(\mu_{s})-M_{i}^{\delta }\right\|}_{L^{2}(\partial X)}^{2}+\frac{\alpha }{2}{\left\|\mu_{s}-\mu_{s}^{*}\right\|}_{L^{2}(X)}^{2} \end{array}$$

with a specified *μ*_s_^*^ based on* a priori* information on the solution. In the statistical inversion framework, the corresponding prior constructions are known as smoothness priors [36]. To compute a minimizer of problem (14), many iterative regularization methods are available. We use the Levenberg-Marquardt method based on linearization. Specifically, for every 1 ≤ *i* ≤ *s*, the forward operator *F*_*i*_(*μ*_*s*_) is linearized around some initial guess *μ*_*s*_^0^; that is,

(16) $$\begin{array}{c} F_{i}\left(\mu_{s}\right)=F_{i}\left(\mu_{s}^{0}\right)+F_{i}^{\prime }\left(\mu_{s}^{0}\right)\left(\mu_{s}-\mu_{s}^{0}\right)+R\left(\mu_{s}^{0};i\right), \end{array}$$

where *F*_*i*_′(*μ*_*s*_^0^) is the* Fréchet* derivative of *F*_*i*_(*μ*_*s*_) with respect to the coefficient *μ*_*s*_ at *μ*_*s*_^0^ and *R*(*μ*_*s*_^0^; *i*) denotes the Taylor remainder for the linearization around *μ*_*s*_^0^. Then substituting the above linearized expression into the functional *J*(*μ*_*s*_) and ignoring the higher-order remainder *R*(*μ*_*s*_^0^; *i*), we get a linearized problem

(17) inf⁡μs∈D 12∑i=1sFiμs0+Fi′μs0μs−μs0−MiδL2∂X2mm+α2μs−μs0L2(X)2.

The Euler equation of the discrete problem is

(18) ∑i=1sFi′μs0∗Fi(μs0)+Fi′(μs0)(μs−μs0)−Miδmm+α(μs−μs0)=0;

that is,

(19) ∑i=1sFi′μs0∗Fi′(μs0)+αI(μs−μs0)m=−∑i=1sFi′μs0∗Fiμs0−Miδ

with *I* the identity matrix. The system can be solved directly to get a new estimate for *μ*_*s*_ based on the initial guess *μ*_*s*_^0^. Then we iteratively update the reconstruction by taking the solution as an initial guess. In practice, the iterative procedure achieves required accuracy within a few iterations. The complete algorithm is given in Algorithm 1. The stopping criterion can be defined based on monitoring the relative change of consecutive iterations.

### 3.2. Sparsity Reconstruction

In the sparsity reconstruction, the functional to be minimized is of the form

(20) $$\begin{array}{c} J\left(\mu_{s}\right)=\frac{1}{2}\overset{s}{\underset{i=1}{\sum }}{\left\|F_{i}(\mu_{s})-M_{i}^{\delta }\right\|}_{L^{2}(\partial X)}^{2}+\frac{\alpha }{2}{\left\|\mu_{s}\right\|}_{l_{1}}. \end{array}$$

We will apply the Bregman framework to solve (20). The key to our method is to “decouple” the *L*_1_ and *L* ^2^ portions in (20). Rather than (20), we will consider the constrained problem [23]:

(21) $$\begin{array}{c} \underset{\mu_{s},d\;}{\inf}\frac{1}{2}\overset{s}{\underset{i=1}{\sum }}{\left\|F_{i}(\mu_{s})-M_{i}^{\delta }\right\|}_{L^{2}(\partial X)}^{2}+\alpha {\left\|d\right\|}_{l_{1}}\;\text{such}\;\;\text{that}\;\;d=\mu_{s}. \end{array}$$

To solve the above minimization problem, the corresponding unconstrained optimization problem is

(22) μs=argmin⁡μs12∑i=1sFi(μs)−MiδL2(∂X)2+αμsl1M+β2d−μsl22,

where *β* > 0 is the split parameter. Then we can iteratively solve the following subproblems [37, 38]:

(23) μsk,dk=argmin⁡μs,d12∑i=1sFi(μs)−MiδL2(∂X)2+αdl1M+β2d−μs−bdk−122,bdk=bdk−1+μsk−dk.

The minimization of the above subproblems can be iteratively solved by splitting it into the minimizations of *μ*_*s*_ and *d* separately. This suggests the following steps.

**Step 1 .**

Consider

(24) μsk=argmin⁡μs12∑i=1sFiμs−MiδL2∂X2M+β2dk−1−μs−bdk−122.

**Step 2 .**

Consider

(25) $$\begin{array}{c} d^{k}=\arg\underset{d}{\min}{\left\|d\right\|}_{l_{1}}+\frac{\beta }{2}{\left\|d-\mu_{s}^{k}-b_{d}^{k-1}\right\|}_{2}^{2}. \end{array}$$

**Step 3 .**

Consider

(26) $$\begin{array}{c} b_{d}^{k}=b_{d}^{k-1}+\mu_{s}^{k}-d^{k}. \end{array}$$

For the solving of Step 1, we can use an iterative method, for example, the Landweber iteration method, the Levenberg-Marquardt method, and so forth. Since the Levenberg-Marquardt method has a higher convergence rate than the conventional Landweber iteration method, we use the Levenberg-Marquardt method on Step 1. Thus, we will solve a minimization problem as follows.

*Step  1* ^*^. Consider

(27) μsk=argmin⁡μs12∑i=1sFi(μsk−1)+Fi′(μsk−1)(μs−μsk−1)−Miδ22M+β2dk−1−μs−bdk−122.

To solve Step  1^*^, we solve the explicitly given variational equation as follows:

(28) ∑i=1sFi′μsk−1∗Fi′(μsk−1)+βI(μs−μsk−1)=βdk−1−μsk−1−bdk−1+∑i=1sFi′μsk−1∗Fiμsk−1−Miδ.

Step 2 is an *L*_1_ norm regularization problem and it can be solved efficiently through the shrinkage operator; that is,

(29) $$\begin{array}{c} d^{k}=\text{shrink}\left(\mu_{s}^{k}+b_{d}^{k-1},\frac{\alpha }{\beta }\right), \end{array}$$

where the shrinkage operator

(30) $$\begin{array}{c} \text{shrink}\left(x,t\right)=\mathrm{sign}\left(x\right)\max\left(\left|x\right|-t,0\right)=\left\{\begin{array}{cc} x-t, & x\geq t,\\ 0, & |x|<t,\\ x+t, & x\leq -t. \end{array}\right. \end{array}$$

From the three steps, we can see that the speed of the split Bregman method is largely dependent on the speed of solving Step 1, where the computation of the Jacobian matrix *F*_*i*_′(*μ*_*s*_) is very time-consuming. We list the split Bregman method in Algorithm 2.

### 3.3. Analysis of Standard Reconstruction and Sparsity Reconstruction

The appropriate choice of regularization depends on* a priori* knowledge of the solutions. Although *L*_2_-norm regularization is a natural choice for its simplicity, it is not always the optimal strategy.

For the DOT reconstruction, the sought-for optical coefficients distribution usually consists of an essentially uninteresting background plus some small inclusions. Thus we will require the solution of the reconstruction in a sparse coefficient vector form; that is, the coefficient vector of the optical parameter contains only a finite number of nonzero elements.

**Figure 1:**
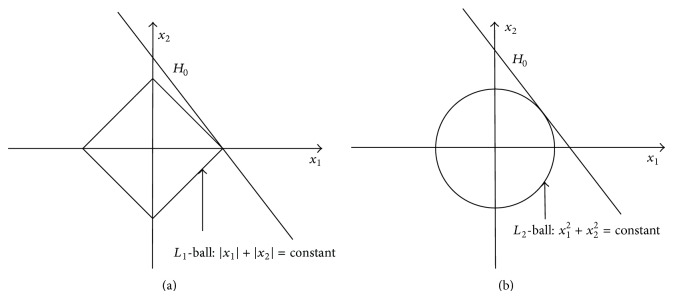
*L*_1_ regularization and *L*_2_ regularization.

The standard reconstruction and sparsity reconstruction add *L*_2_-norm penalization and *L*_1_-norm penalization into the problem, respectively, which allow additional constraints or prior information towards the approximate solution.  Figure 1(a) illustrates how *L*_1_-norm penalization leads to sparse solutions. Take a linear problem as an example; suppose we are looking for a solution of the linear equation *Ax* = *y* in a model space with two degrees of freedom and the line *H*_0_ consists of *x* that satisfy *Ax* = *y*. To find the solution with smallest *L*_2_-norm, we can imagine taking a small circle around the origin and increase its radius until it first touches the solution line *H*_0_: the tangent point is the minimum; see Figure 1(b). Obviously, the point of solution has both *x*_1_ and *x*_2_ components. In a similar way, to find the solution with smallest *L*_1_-norm, we take a small *L*_1_-ball around the origin and increase its radius until it first touches *H*_0_: the touching point is the minimum *L*_1_-norm solution, which is sparser than the solution achieved with smallest *L*_2_-norm, because it is on one axis only; only one component is nonzero.

There is also an extreme penalty term, say, *L*_0_-norm penalization, which is simply the number of nonzero coefficients and leads to the sparsest solution. Nevertheless, the optimization problem with this penalty is not computationally tractable. Hence *L*_1_-norm penalization is preferred in practical problems. In addition, it has been proven that, for some large matrices *A*, if there exist sufficiently sparse solutions, the sparsest solution can be achieved by the *L*_1_-norm minimization [19, 20]. Under certain conditions, the *L*_1_ penalty term can provide an accurate result even with limited observations [17]. These phenomena are further investigated in different fields such as electrical impedance tomography and tomographic inversion [39, 40].

## 4. Numerical Implementations

In this section, we report simulation results on numerical examples in two dimensions (2D). We perform the simulations on a 3.0 GHz PC with 8 GB RAM in MATLAB 2013b environment under Windows 7. In our simulations, the scattering coefficient *μ*_*s*_ is reconstructed both with the standard regularization reconstruction and with the sparsity regularization reconstruction. We use two kinds of measurements for reconstruction: boundary angular-averaged measurements and internal angular-averaged measurements. The boundary measurements are the excitance received by detectors attached to the boundary of tissue. Due to the limit of measurement techniques of the optical devices, we cannot accurately receive the excitance from all angles; instead we receive a boundary angular-averaged data ∫_*Ω*^+^_ *u*(**x**, **ω**)*dσ*(**ω**) which can be regarded as the integration of radiance on the boundary of the domain in all outgoing directions. The internal angular-averaged measurements are the same as boundary angular-averaged measurements except for the location of detectors which we assumed to be inside the domain.

We design different simulations to demonstrate the following points.

First, for sparse coefficient distribution, the sparsity regularization reconstruction localizes the location of the inclusion better than standard regularization reconstruction, especially when there is noise and the data are incomplete. See Figures 4, 9, and 10.

Second, the proposed sparsity regularization reconstruction works better when *g* = 0.9 than *g* = 0.1. See Figures 6 and 7.

Third, when the internal angular-averaged measurements are available, the proposed sparsity regularization reconstruction works better with internal angular-averaged measurements than with boundary angular-averaged measurements. See Figures 13 and 14.

Last, the split Bregman algorithm can efficiently solve the DOT image reconstruction problem with sparsity regularization. The results reconstructed by the Bregman algorithm are more accurate than those achieved by three other algorithms. See Figures 16 and 17.

We mention here that, in the DOT reconstruction problems, the measurement data are usually synthesized from the numerical solution of the forward problem. Under this situation, the phenomenon of inverse crime will happen especially when the same discretization is used for the forward and inverse process, because it will make the ill-posedness of the inverse problem not evident [41, 42]. Hence, in order to avoid the inverse crime, we will use different discretization meshes in the forward and inverse problems.

Now let us state our numerical experiments. Before stating the details of every experiment, we first state some common experiment settings that will be used in all of our experiments. Our purpose is to reconstruct the scattering coefficient *μ*_*s*_ based on the BVP (2) and (3), where we assume that the following 2D simulations are all performed on a unit circular domain centered at (0 mm, 0 mm), corresponding to the the internal light source *f* = 0, the inflow current for every incident impulse *u*_in,*i*_ that is settled as

(31) $$\begin{array}{c} u_{\text{in},i}=B_{i},\;i=1,\ldots ,s. \end{array}$$

The boundary value *B*_*i*_ is a piecewise linear function whose spatial support is *S*_*i*_ and achieves the value 1 at the center node of *S*_*i*_; here *S*_*i*_ denotes the finite element through which the *i*th incident impulse passes. The direction of the *i*th incident impulse **ω**_*i*_ is node of the angular mesh that points approximately from the center of *S*_*i*_ to the center of *X*.

If we denote the domains that contain the inclusions as *X*_0_, then the background domain is *X*_1_ = *X*∖*X*_0_. Then the true value of absorption coefficient *μ*_*a*_(**x**) in BVP (2) and (3) is defined as

(32) $$\begin{array}{c} \mu_{a}\left(x\right)=\left\{\begin{array}{cc} 0.1, & x\in X_{1},\\ 0.01, & x\in X_{0}. \end{array}\right. \end{array}$$

Since the absorption coefficient is assumed to be known in our scattering reconstruction problems, we only give the mathematical formulation of the absorption coefficient. While in order to compare explicitly the true value and the reconstructed value of scattering coefficient, we demonstrate the true value and the reconstructed results of scattering coefficient in figures.

**Figure 2:**
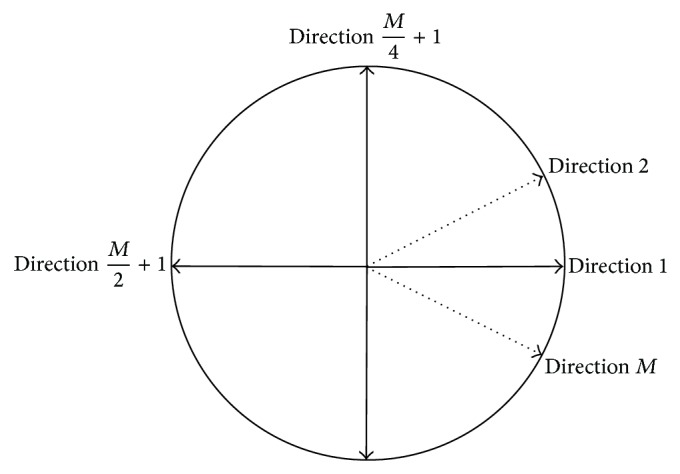
Angular discretization in 2D.

In order to make discretization in angle, we can divide the angular space [0,2*π*) uniformly into *M* directions with equal interval length as shown in Figure 2. And we set *M* = 32 in all examples; that is, there are 32 angular directions.

**Figure 3:**
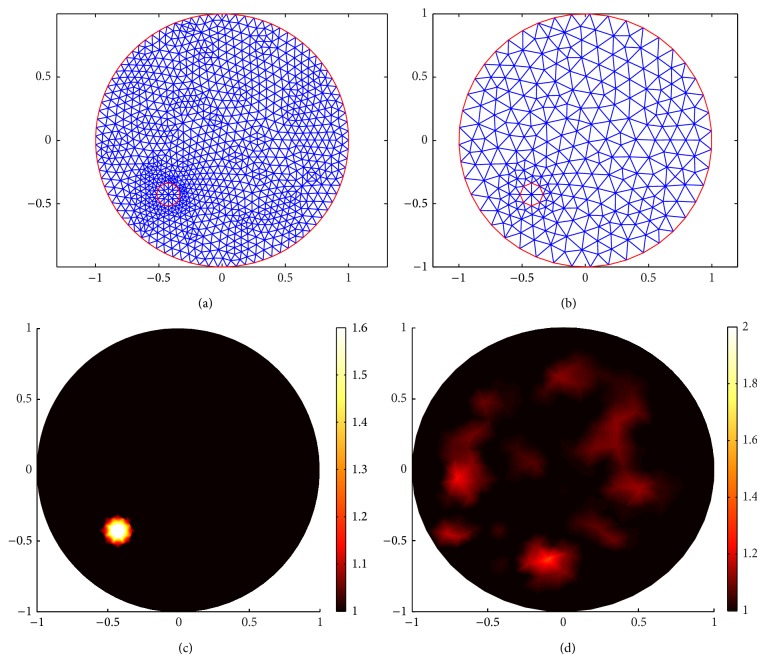
Standard regularization reconstruction for a small spot with 132 boundary angular-averaged measurements. (a) Mesh for the forward problem, (b) mesh for the inverse problem, (c) the true scattering distribution, and (d) the image reconstructed from standard regularization.

In the first example, Figure 3, a small inclusion with 0.1 mm diameter, is centered at (−0.43, −0.43). 12 sources and 12 detectors are equally spaced attached to the boundary of the circle domain. The simulated boundary angular-averaged measurements performing on the boundary are generated with a spatial mesh (a) of 1097 nodes and 2104 elements and reconstruction mesh (b) having 286 nodes and 526 elements.

**Figure 4:**
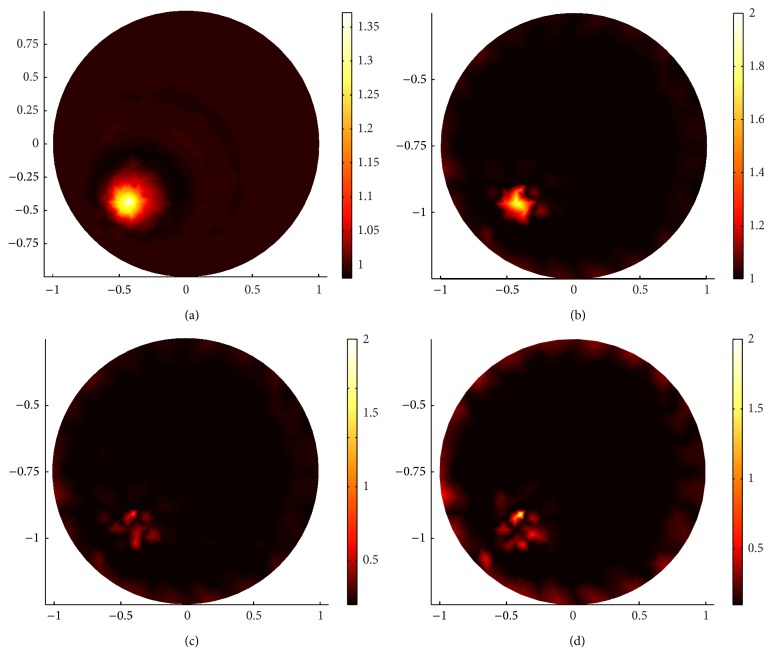
Sparsity regularization reconstruction for a small spot with 132 boundary angular-averaged measurements, images with (a) 0%, (b) 0.1%, (c) 1%, and (d) 10% Gaussian noise, respectively.

The image from standard regularization reconstruction without noise is displayed in (d) in Figure 3. The images from sparsity regularization reconstruction without or with noise are displayed in Figure 4 with the noise defined as *M*_*i*_^*δ*^ = *M*_*i*_(1 + *δN*), where *δ* is the signal-to-noise ratio and *N* is a Gaussian random variable with zero mean and unity variation. In Figure 4, we vary *δ* from 0 percent to 10 percent and plot the sparsity regularization reconstruction image. Comparing images from standard regularization and sparsity regularization, we conclude that sparsity regularization can efficiently localize the sparse inclusion with a few measurements, even in the presence of different degrees of noise, while standard regularization fails to reconstruct the sparse inclusion even without noise.

In the second example, we compare the sparsity regularization reconstruction and the standard regularization reconstruction on squares of three different sizes.

**Figure 5:**
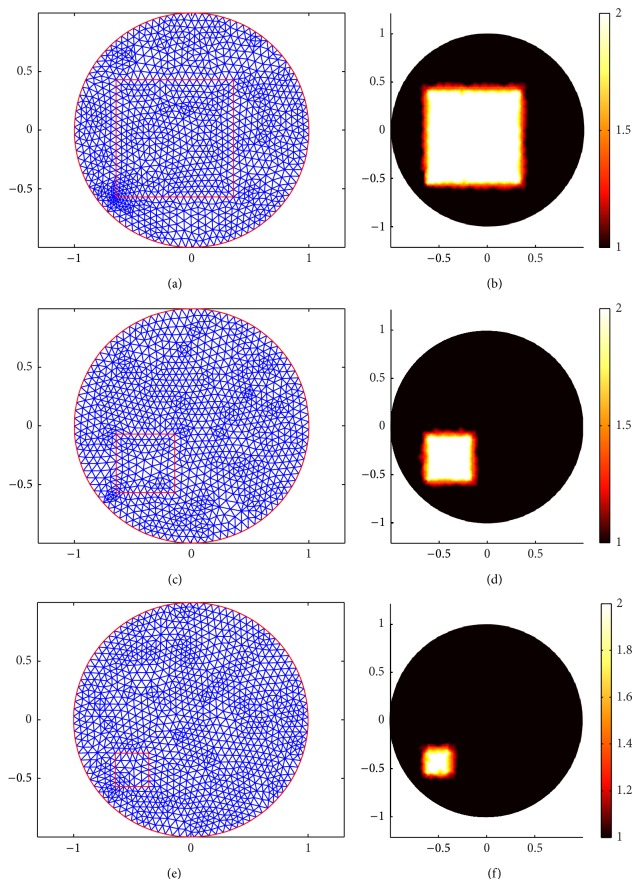
Meshes for square cases. (a), (c), and (e) are forward meshes for three square cases, respectively. (b), (d), and (f) are the true scattering coefficient distribution for big square case, middle square case, and small square case, respectively.

In Figure 5, (a), (c), and (e) show the forward mesh of three different sizes squares. (b), (d), and (f) show the true distribution of the scattering coefficient in three different experiments.

**Figure 6:**
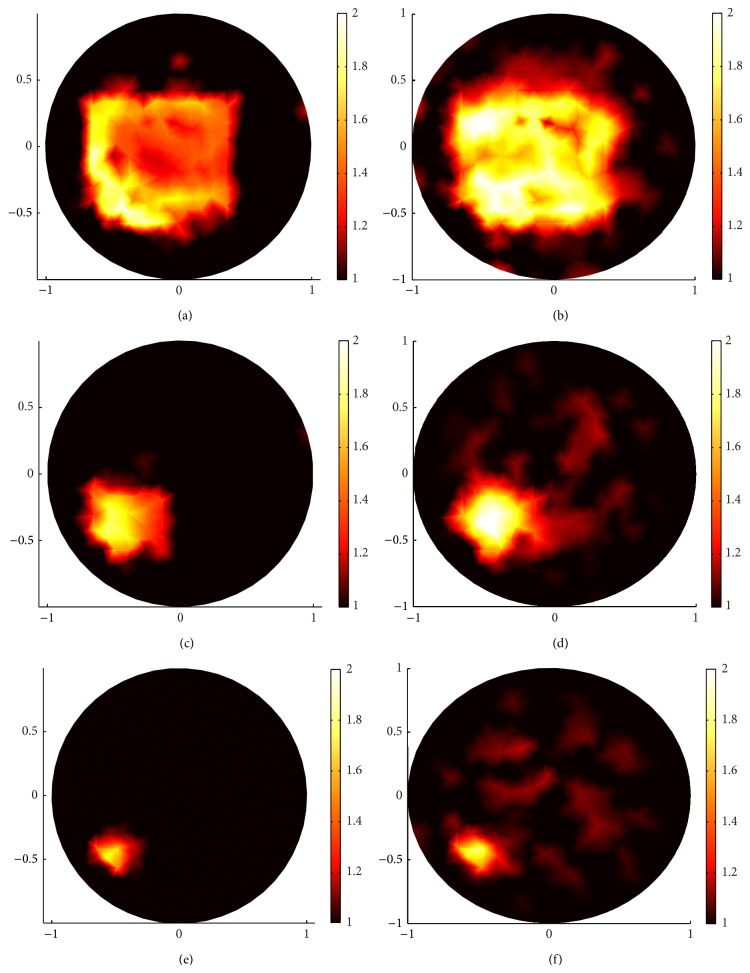
Comparison of sparsity regularization reconstruction and standard regularization reconstruction with 132 boundary angular-averaged measurements when *g* = 0.1. (a), (c), and (e) are reconstructed images with sparsity regularization for big square, middle square, and small square, respectively. (b), (d), and (f) are reconstructed images with standard regularization for big square, middle square, and small square, respectively.

**Figure 7:**
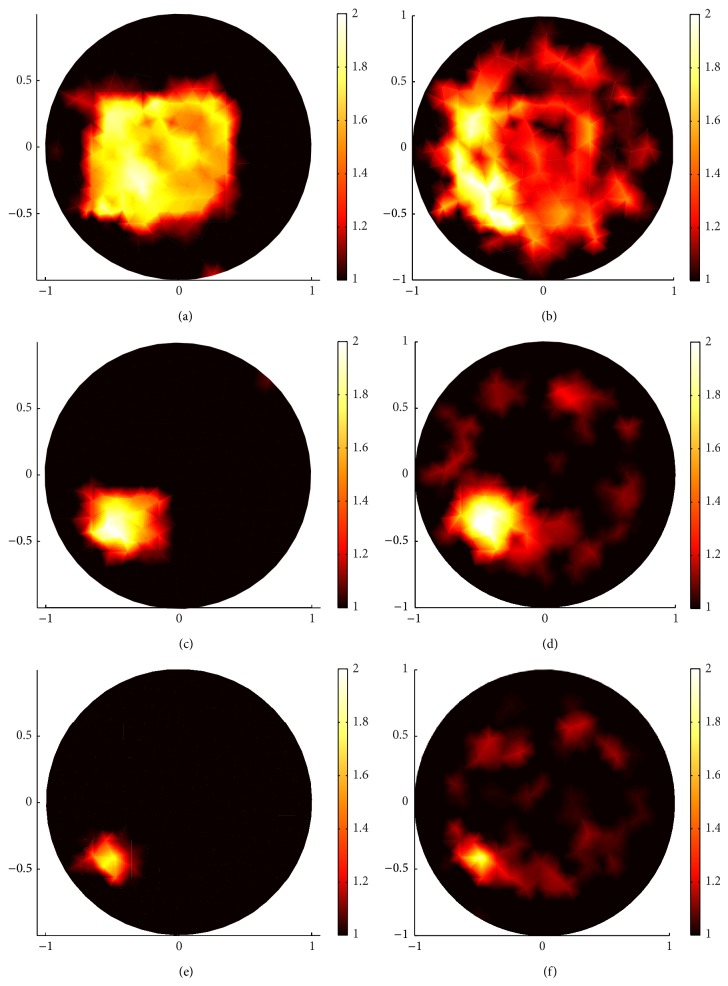
The same reconstructions as in Figure 6 but with *g* = 0.9. (a), (c), and (e) are reconstructed images with sparsity regularization for big square, middle square, and small square, respectively. (b), (d), and (f) are reconstructed images with standard regularization algorithm for big square, middle square, and small square, respectively.

Comparing (a), (c), and (e) in Figures 6 and 7 and (b), (d), and (f) in Figures 6 and 7, clearly, the sparsity regularization can localize the position of the inclusion better than the standard regularization in all the three kinds of squares and has a clear contrast with the backgrounds. But the standard regularization also has its advantages. Comparing (a) and (b), (c) and (d), and (e) and (f) in Figure 6, when *g* = 0.1, there are many blurred dots in the standard regularization reconstructed images; we can not identify the distribution domain of the reconstructed scattering coefficient, but the value of the scattering coefficient in standard regularization reconstruction is much closer to the true value of the scattering coefficient than that in the sparsity regularization reconstruction. However, this advantage is broken when *g* = 0.9; see Figure 7.

On the other hand, the proposed sparsity regularization reconstruction performs better when *g* = 0.9 than when *g* = 0.1 in localizing the position of the inclusions as well as identifying the value of the scattering coefficient.

In conclusion, when the anisotropic factor is small, the standard regularization can identify the value of the scattering coefficient better than the sparsity regularization. Although there are some blurred dots in standard regularization reconstructed images, one can remove them by using multilevel approach. On the other hand, the sparsity regularization can localize the position of the inclusion better than standard regularization and has a clear contrast, especially in the forward-peaking regime with big anisotropic factor *g*.

**Figure 8:**
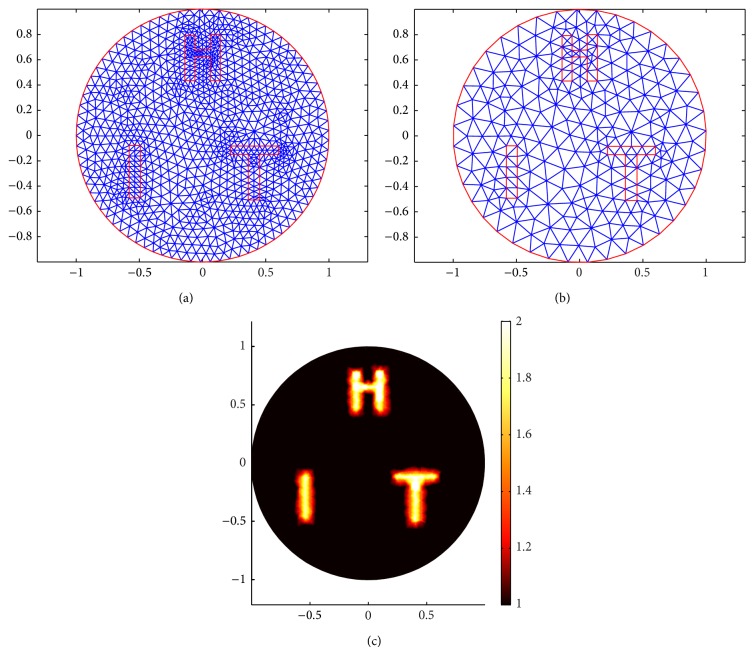
Meshes for three-letter case. (a) is the forward mesh. (b) is the inverse mesh. (c) is the true scattering coefficient distribution.

In the third example (Figure 8), three-letter inclusions (a) are put in the domain to evaluate our reconstruction algorithms; the forward mesh (a) has 1077 nodes and 2072 elements; the reconstruction mesh (b) has 280 nodes and 518 elements.

**Figure 9:**
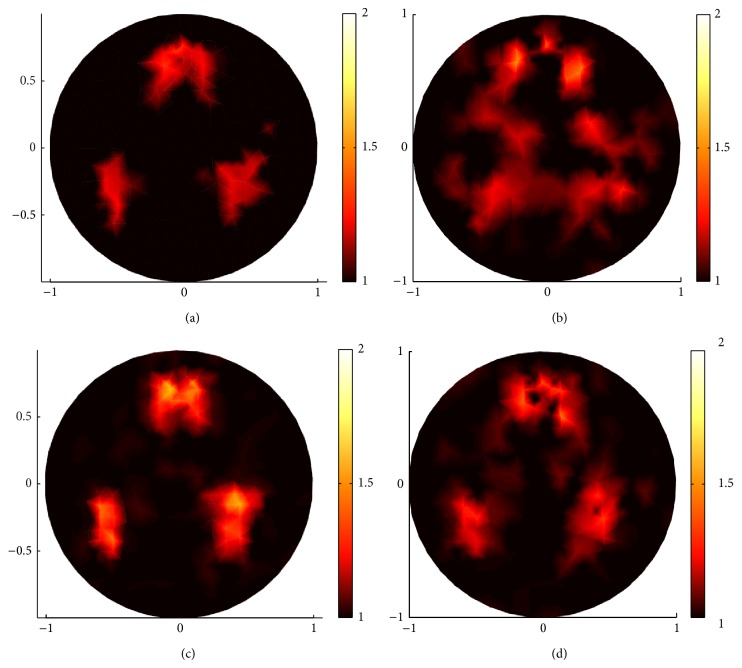
Reconstruction with boundary data when *g* = 0.1. (a) and (c) are reconstructed images by sparsity regularization with 132 boundary angular-averaged measurements and 380 boundary angular-averaged measurements. (b) and (d) are reconstructed images by standard regularization with 132 boundary angular-averaged measurements and 380 boundary angular-averaged measurements.

**Figure 10:**
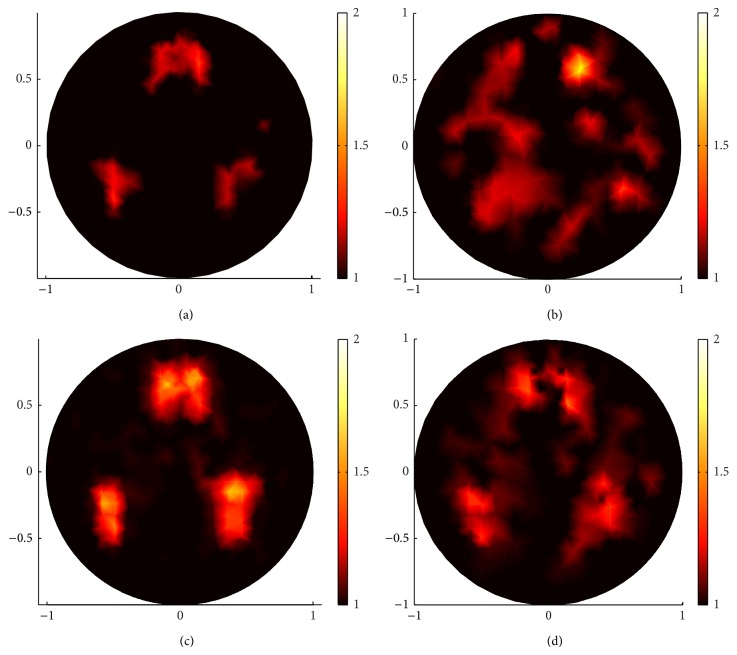
The same reconstructions as in Figure 9 but with *g* = 0.9. (a) and (c) are reconstructed images by sparsity regularization with 132 boundary angular-averaged measurements and 380 boundary angular-averaged measurements. (b) and (d) are reconstructed images by standard regularization with 132 boundary angular-averaged measurements and 380 boundary angular-averaged measurements.

In Figures 9 and 10, the reconstructions with *g* = 0.1 and 0.9, respectively, are carried out to demonstrate that sparsity regularization ((a), (c) in Figures 9 and 10) in general reconstructs better images than standard regularization ((b), (d) in Figures 9 and 10).

Comparing (a) and (c) in Figures 9 and 10, respectively, we find that the proposed sparsity regularization reconstruction performed better when the inverse problem is more severely ill-posed due to fewer measurements.

In conclusion, efficient algorithm and proper regularization, for example, sparsity regularization reconstruction for sparse coefficient distribution, are essential to recover high-resolution images. On the other hand, we can notice that there are blurred dots in the middle of the reconstructed images in (a) and (c) in Figures 9 and 10, due to the ill-posedness of inverse problem. These blurred dots have small contrast and thus can be removed through sparsity regularization reconstruction by choosing proper *α* and *β* or, beyond our paper, through multilevel approach [43].

Next, using sparsity regularization reconstruction, we will show the superiority of the internal angular-averaged measurements over the boundary angular-averaged measurements with two relatively complicated cases. The detectors for internal angular-average measurements in two cases lie on a 0.95-radius circle equidistant from the center at (0 mm, 0 mm). Case 1 is shown in the first column in Figures 13 and 14; Case 2 is shown in the second column in Figures 13 and 14.

**Figure 11:**
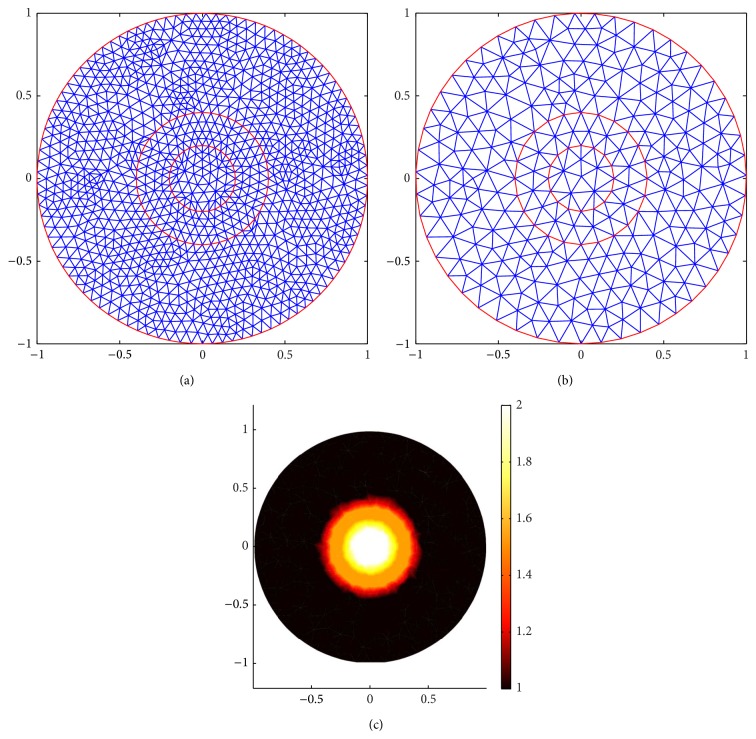
Meshes for Case 1. (a) is the forward mesh. (b) is the inverse mesh. (c) is the true scattering coefficient distribution.

**Figure 12:**
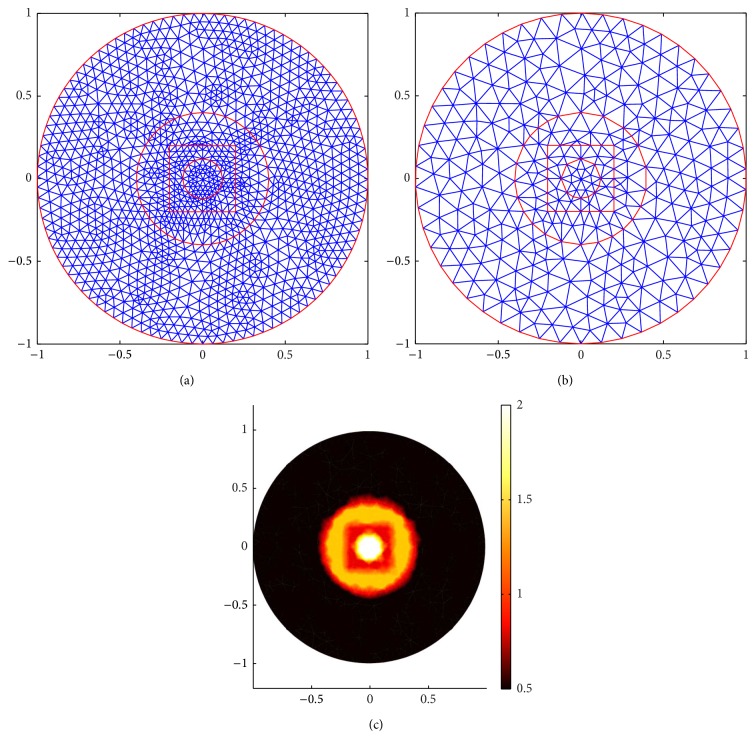
Meshes for Case 2. (a) is the forward mesh. (b) is the inverse mesh. (c) is the true scattering coefficient distribution.

Figure 11 shows the meshes for Case 1. (a) shows the forward mesh; it has 1131 nodes and 2160 elements. (b) shows the inverse mesh; it has 296 nodes and 540 elements. (c) shows the true distribution of the scattering coefficient.  Figure 12 shows the meshes for Case 2. (a) shows the forward mesh; it has 1347 nodes and 2592 elements. (b) shows the inverse mesh; it has 350 nodes and 648 elements.

**Figure 13:**
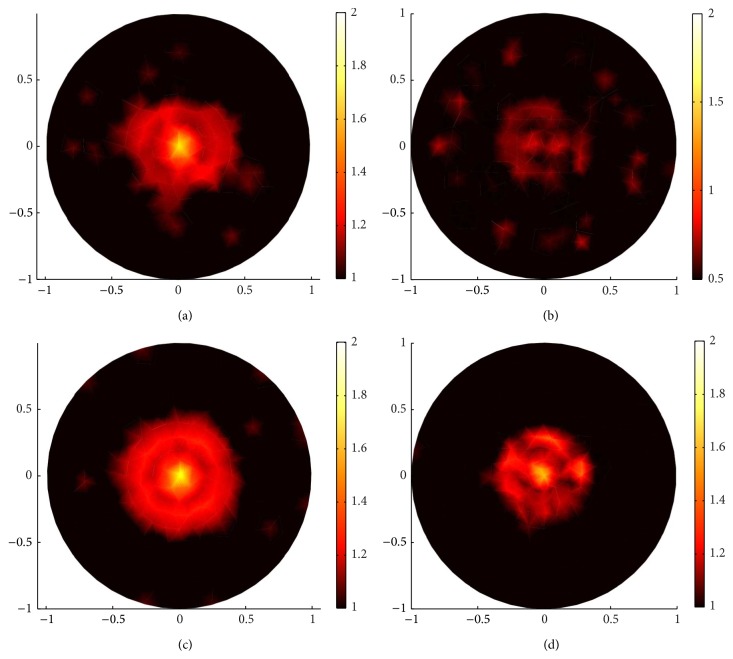
Sparsity regularization reconstruction when *g* = 0.1. (a) and (c) are reconstructed images for Case 1 with 132 boundary angular-averaged measurements and 132 internal angular-averaged measurements, respectively. (b) and (d) are reconstructed images for Case 2 with 240 boundary angular-averaged measurements and 240 internal angular-averaged measurements, respectively.

**Figure 14:**
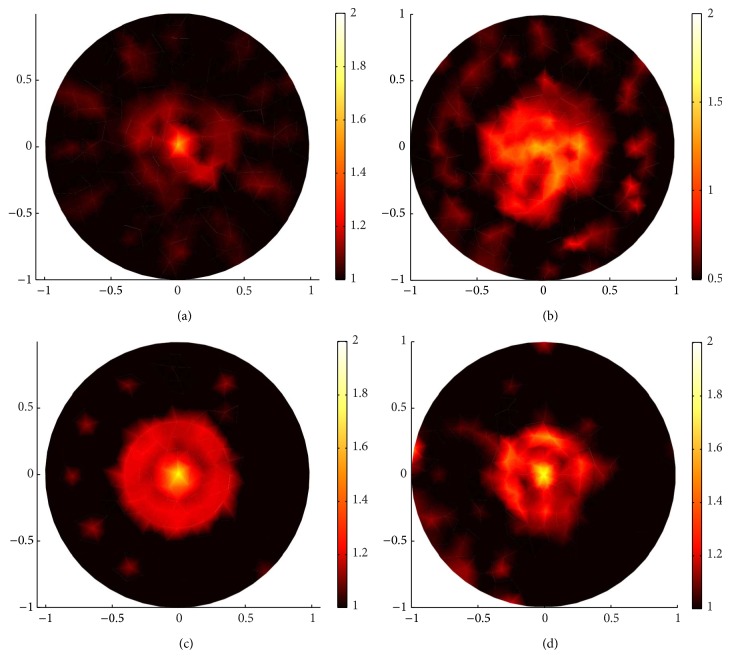
The same reconstructions as in Figure 13 but with *g* = 0.9. (a) and (c) are reconstructed images for Case 1 with 132 boundary angular-averaged measurements and 132 internal angular-averaged measurements, respectively. (b) and (d) are reconstructed images for Case 2 with 240 boundary angular-averaged measurements and 240 internal angular-averaged measurements, respectively.

Comparing (a) and (c) in Figures 13 and 14, we find that, for Case 1, the internal angular-averaged measurements ((c) in Figures 13 and 14) can localize the position of the outside inclusion, although the internal inclusion is localized blurred while the boundary angular-averaged measurements ((a) in Figures 13 and 14) can not completely localize any inclusion.

For the complicated Case 2 ((b) and (d) in Figures 13 and 14), we increase the measurement data by increasing the number of detectors to 16, respectively, in order to get more information from the boundary measurements. Both of the two kinds of measurements can not accurately identify the inclusion, but the internal angular-averaged measurements ((d) in Figures 13 and 14) performed relatively better than the boundary angular-averaged measurements ((b) in Figures 13 and 14). A justification of this phenomenon is that the energy of the incident current is decayed much due to the large and relatively frequently change of the scattering coefficient of the inclusion. One can alleviate this phenomenon by increasing the number of detectors or measurement data. In conclusion, the internal data can better-pose the inverse problem.

**Figure 15:**
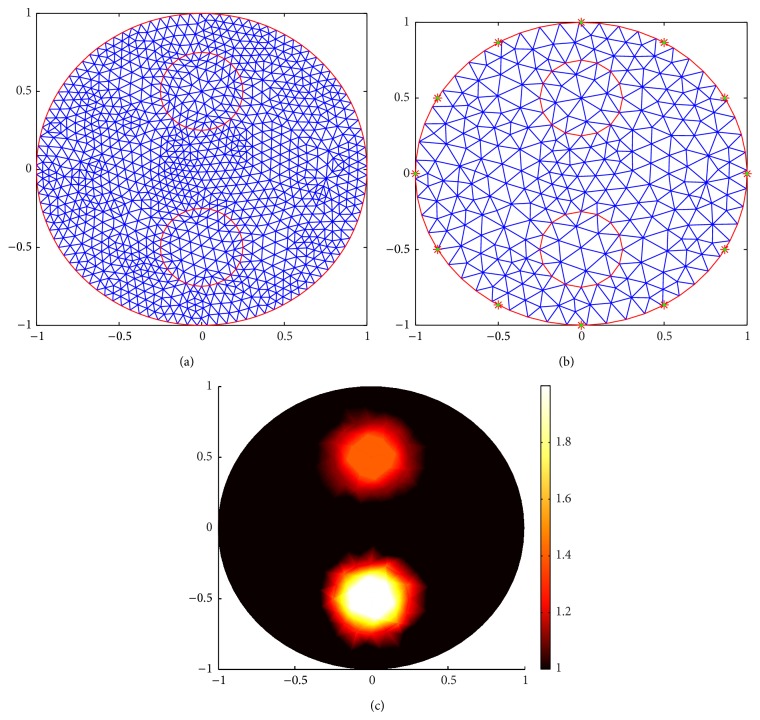
Meshes for Case 3. (a) is the forward mesh. (b) is the inverse mesh. (c) is the true scattering coefficient distribution.

As the last simulation, we compute Case 3 in Figure 15 to investigate the performance of split Bregman algorithm for DOT image reconstruction with sparsity regularization. The results are compared with Gauss-Newton algorithm [44] and the state-of-the-art *L*_1_ algorithms including GPSR [45] and YALL1 [46]. To deal with the nondifferentiability of the absolute value |*x*| at *x* = 0 in Gauss-Newton algorithm, we replace |*x*| by $|x|=\sqrt{x^{2}+\gamma }$,  *γ* > 0. We choose *γ* = 1*e* − 6 in Gauss-Newton algorithm.

**Figure 16:**
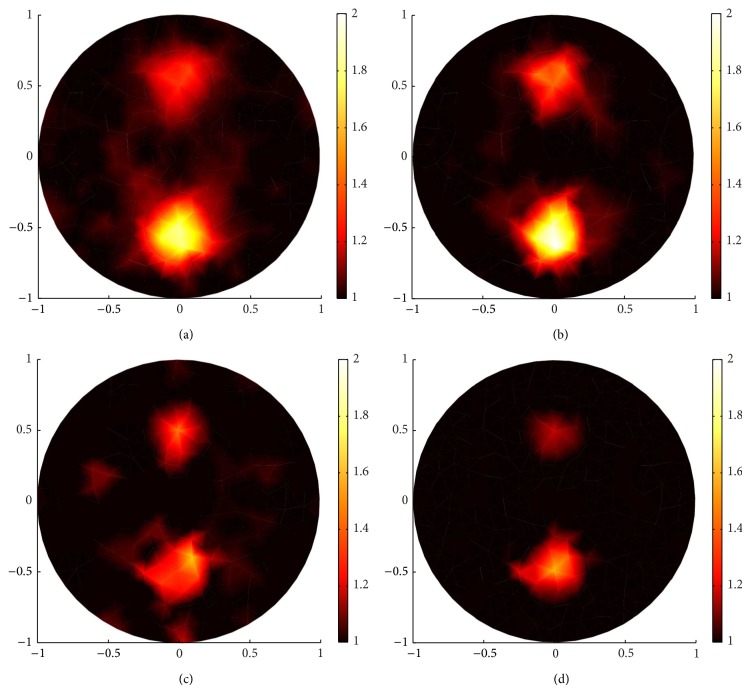
Sparsity regularization reconstruction when *g* = 0.9 with the measurement noise level of 0.1% and 132 boundary angular-averaged measurements, using four different algorithms. (a)–(d) are reconstruction results by the split Bregman, YALL1, GPSR, and Gauss-Newton algorithm, respectively.

**Figure 17:**
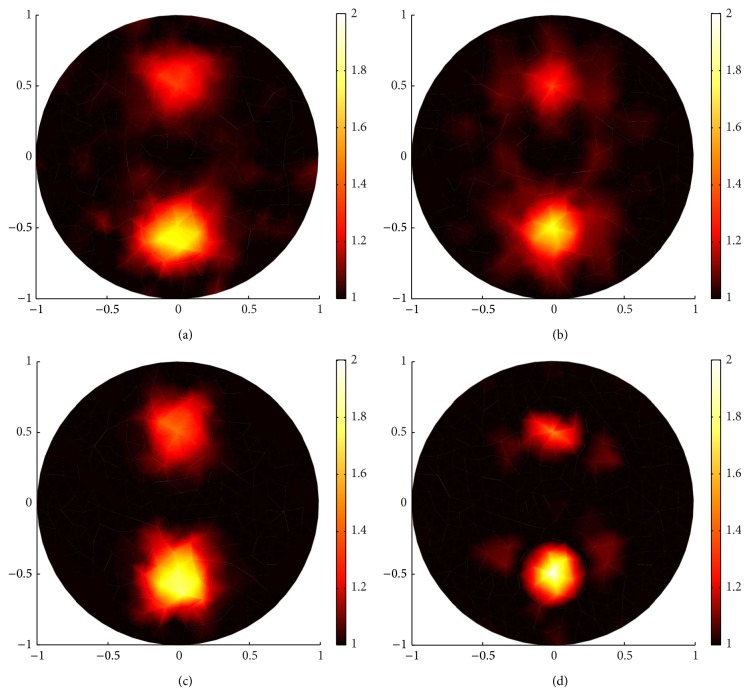
Sparsity regularization reconstruction when *g* = 0.1 with the measurement noise level of 0.1% and 132 boundary angular-averaged measurements, using four different algorithms. (a)–(d) are reconstruction results by the split Bregman, YALL1, GPSR, and Gauss-Newton algorithm, respectively.

In Case 3, two circle inclusions are embedded at the top and bottom of the circle domain with 1137 nodes and 2168 elements for the forward mesh and 298 nodes and 542 elements for the inverse mesh. We use 132 boundary angular-averaged measurements with 0.1% Gaussian noise for reconstruction. The reconstructed images are shown in Figures 16 and 17, and we summarize the results with *g* = 0.9 in Table 1, containing the parameters for the four methods, computational time, data misfit ∑_*i*=1_^*s*^‖*F*_*i*_(*μ*_*s*_) − *M*_*i*_^*δ*^‖_*L*^2^(∂*X*)_^2^, relative solution error norm (RE), and signal-to-noise ratio (SNR). The relatively optimal parameters are chosen empirically.

The RE is calculated as

(33) $$\begin{array}{c} \text{RE}=\frac{{\left\|\mu_{s}-\mu_{s}^{\text{true}}\right\|}_{2}}{{\left\|\mu_{s}^{\text{true}}\right\|}_{2}}, \end{array}$$

and SNR is calculated as

(34) $$\begin{array}{c} \text{SNR}=10\;\text{lo}{\text{g}}_{10}\frac{{\left\|\text{signal}\right\|}_{2}}{{\left\|\text{noise}\right\|}_{2}}. \end{array}$$

The reconstruction results in Figures 16 and 17 show that all of the four algorithms can locate the position of two inclusions. The split Bregman and GPSR algorithm can achieve more accurate shape of the inclusions, and the reconstructed scattering coefficients achieved by the two algorithms are closer to the true value. The Gauss-Newton algorithm can find the approximate location of the inclusions, but the reconstructed circle inclusion is smaller in size and the reconstructed scattering coefficient departs from actual ones.

The results displayed in Table 1 also demonstrate that the split Bregman algorithm performs better. It leads to the lowest RE and data misfit with less calculation. The GPSR algorithm achieves higher SNR at the expense of about 25% extra computational time. The Gauss-Newton algorithm spends least computational time, but the reconstruction error is not very satisfactory.

These results are justified by the fact that the split Bregman algorithm decouple the sparsity reconstruction problem into *L*_1_ and *L*_2_ portions leading to a better compromise in the efficiency and quality of the reconstructed optical parameters.

## 5. Conclusion

In this paper, by using the image modalities in DOT, we employ the sparsity regularization method on the RTE-based coefficient identification problems, which is proven to perform in general better than the standard regularization reconstruction, especially for sparse distribution coefficient and large noise, and in the forward-peaking regime with big anisotropic factor *g*. On the other hand, we construct cases and compare the reconstruction results with boundary and internal measurement to test the validity of the proposed method; results show that the proposed method is practicable and feasible; it performs steadily with various measurement data; meanwhile, the internal measurement can better-pose the inverse problem and achieve more accurate results. However, we usually cannot obtain the internal measurement data in practice. Hence, our method cannot reconstruct inclusions with complicated internal structure accurately with small amount of boundary angular-averaged measurements. One can alleviate this phenomenon by increasing the number of detectors or measurement data. In the further work we will consider seeking for multi-imaging modality which can further improve the inversion quality with boundary angular-averaged measurements. Results show the competitive performance of the split Bregman algorithm for the DOT image reconstruction compared with other existing *L*_1_ algorithms.

## Figures and Tables

**Algorithm 1:**
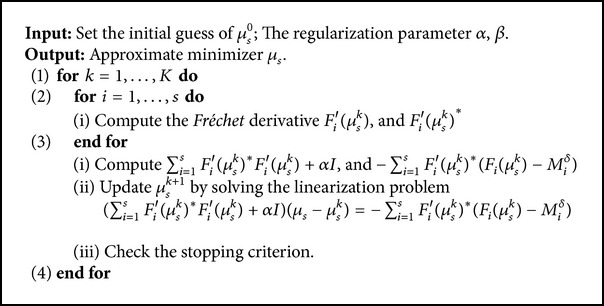
Reconstruction algorithm based on the regularizing Levenberg-Marquardt method.

**Algorithm 2:**
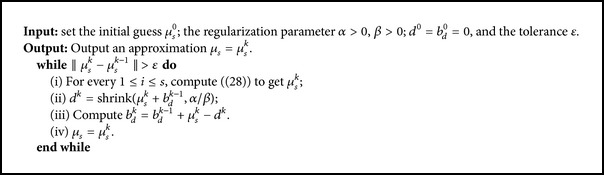
Reconstruction algorithm based on the split Bregman method.

**Table 1:** Comparison of sparsity reconstructions for Case 3 when *g* = 0.9 with the regularization parameter *α* = 1*e* − 5, *β* is the split parameter of split Bregman method, *τ* represents the tolerance for stopping criterion of YALL1 and GPSR method, and *λ* is the control parameter of Gauss-Newton method.

Algorithms	Split Bregman	YALL1	GPSR	Gauss-Newton
Parameter	*β* = 1*e* − 06	*τ* = 1*e* − 8	*τ* = 1*e* − 8	*λ* = 1*e* − 04
CPU time (s)	79.426	90.761	101.321	75.197
Data misfit	0.0044	0.0120	0.0046	0.03087
RE	0.1464	0.2252	0.1624	0.2141
SNR	4.9827	4.6311	5.2010	4.3402
